# Knowledge, attitude, and practice toward family-based treatment among parents of children with leukemia

**DOI:** 10.3389/fpubh.2024.1481122

**Published:** 2024-11-25

**Authors:** Xue Yang, Shihua Long, Feng Lu, Zhigui Ma

**Affiliations:** ^1^Department of Pediatrics, West China Second University Hospital, Sichuan University, Chengdu, Sichuan, China; ^2^Key Laboratory of Birth Defects and Related Diseases of Women and Children, Sichuan University, Ministry of Education, Chengdu, Sichuan, China; ^3^Department of Pediatrics Nursing, West China Second University Hospital, Sichuan University/West China School of Nursing, Sichuan University, Chengdu, Sichuan, China

**Keywords:** knowledge, attitude, practice, leukemia, family-based treatment, cross-sectional study

## Abstract

**Background:**

To investigate the KAP toward family-based treatment among parents of children with leukemia.

**Methods:**

This cross-sectional study was conducted between December, 2022 and July, 2023 in the Pediatric hematologic oncology department of West China Second University Hospital, Sichuan University. The study population consisted of parents of children diagnosed with leukemia. Their demographic characteristics and KAP toward family-based treatment for leukemia were collected by self-administered questionnaires.

**Results:**

A total of 482 parents participated, including 379 (78.63%) females, with an average age of 35.83 ± 6.40 years. The mean scores for KAP were 7.28 ± 1.13 (possible range: 0–10), 37.82 ± 4.38 (possible range: 9–45), and 40.09 ± 4.17 (possible range: 9–45), respectively. Multivariate logistic regression analysis indicated that the knowledge score (OR = 1.48, 95% CI: [1.08–2.05], *P* = 0.016), attitude score (OR = 1.31, 95% CI: [1.18–1.46], *P* < 0.001), education of junior college and above (OR = 11.28, 95% CI: [1.94–65.65], *P* = 0.007), and monthly income of 5,000–10,000 Yuan (OR = 10.88, 95% CI: [1.15–102.98], *P* = 0.037) were independently associated with a proactive practice. Structural equation modeling (SEM) results highlighted the significant direct impact of knowledge on attitude (β = 0.72, *P* = 0.002), attitude on practice (β = 0.57, *P* < 0.001), and knowledge on practice (β = 0.81, *P* < 0.001).

**Conclusion:**

Parents of children with leukemia demonstrated inadequate knowledge, but positive attitudes and proactive practices toward family-based treatment for leukemia. Future interventions should not only prioritize augmenting parental knowledge through educational initiatives but also focus on fostering positive attitudes and providing support for both knowledge and practical parenting skills to facilitate proactive involvement.

## Introduction

In developed countries, pediatric cancers, which account for approximately 1% of new cancer diagnoses each year, represent a significant contributor to disease-related mortality among children (1, 2). Among these cancers, the most prevalent types include leukemia, brain tumors, lymphomas, and solid tumors like neuroblastoma and Wilms tumor (3–5). Leukemia, a prevalent form of blood cell cancer, exhibits the highest incidence rate among children and adolescents, impacting approximately 25%−35% of pediatric cancer cases (6). Historically, chemoimmunotherapy has been considered the standard of care for most patients requiring frontline therapy for lymphocytic leukemia or small lymphocytic lymphoma (7). For example, acute lymphoblastic leukemia, accounting for nearly one-third of all childhood cancers, can achieve successful cure through combination chemotherapy alone (8). Following this life-saving procedure, a significant proportion of pediatric patients necessitate an extended course of pharmaceutical intervention and sustained medical oversight. During and after chemotherapy, the active involvement of family caregivers is imperative, as they are tasked not only with shouldering cancer-related responsibilities such as treatment management and symptom monitoring but also with assuming additional daily caregiving duties encompassing tasks such as meal preparation and personal hygiene assistance (9). This underscored the indispensable role of the family in the recovery process, especially within the context of family-based treatment.

The Knowledge, Attitude, and Practice (KAP) survey elucidates a group's comprehension, beliefs, and actions regarding a specific subject, with a specific emphasis on health literacy (10–12). The KAP of parents in the context of childhood leukemia treatment holds paramount importance. These factors exert direct influence on the quality of treatment decisions, emotional support for the child, and the efficacy of everyday caregiving. Accurate knowledge equips parents with an enhanced grasp of treatment alternatives and medication effects, while a positive attitude provides the emotional sustenance essential for the child. Active and informed caregiving practices ensure the proficient implementation of treatment plans, ultimately augmenting the child's quality of life and treatment outcomes. Evaluating the knowledge, attitude, and practices of parents provides valuable insights into their engagement and needs in relation to treatment. These insights can subsequently be used to improve communication between medical teams and families, design more effective family-centered treatment approaches, strengthen parental confidence and skills, and ultimately elevate the care and treatment provided to children with leukemia. Additionally, although preceding research has addressed KAP in connection to childhood leukemia, it has not primarily centered on family treatment for leukemia (13–15). The distinctiveness of this investigation lies in its focal point on the domain of family-based treatment, offering invaluable perspectives and avenues for advancement in future research to better cater to the necessities of both children and their families.

This study aimed to explore the KAP toward family-based treatment among parents of children with leukemia.

## Methods

### Study setting and recruitment

This cross-sectional study was conducted between December, 2022 and July, 2023 at Pediatric hematologic oncology department of West China Second University Hospital, Sichuan University, and included parents of children afflicted with leukemia.

### Inclusion and/or exclusion criteria

The inclusion criteria was Family numbers of children afflicted with leukemia, and the exclusion criteria were: (1) those refuse to participate; (2) invalid questionnaires according to the quality control process.

### Data collection

The questionnaire was self-designed by the research team according to “CSCO: clinical guideline for diagnosis and treatment of leukemias in children and adolescent” and clinical experience. The questionnaire was further refined following feedback from two experts, one with over 20 years of experience in the field of pediatric hematological oncology and the other with over 30 years of experience in the same field. A pilot study was conducted among 42 participants, resulting in a Cronbach's α of 0.867, indicating good internal consistency for the overall questionnaire. The Cronbach's α for knowledge, attitude, and practice subdomains were 0.845, 0.812, and 0.823, respectively.

The final questionnaire was administered in Chinese, and included 4 distinct dimensions. The demographic characteristics section comprised 10 items, including gender, age, residence, education, occupation, monthly income, duration of the child's illness, types of children's diseases, time since the child's treatment, child's age, and guardianships. The knowledge dimension consisted of 10 items, respondents received 1 point for correct answers and 0 points for incorrect or unclear responses, with a possible range of 0–10 points. The attitude dimension included nine items, using a five-point Likert scale, where positive attitude questions (question 1, 3–9) were rated from “strongly agree” (5 points) to “strongly disagree” (1 point). Conversely, the negative attitude question (question 2) was reverse-scored, with a possible score range of 9–45 points. Furthermore, the practice dimension included nine positively-phrased items, using a five-point Likert scale, and were rated from “always” (5 points) to “never” (1 point). Participants achieving scores exceeding 80% of the total, based on Bloom's cutoff, were classified as demonstrating adequate knowledge, a positive attitude, and proactive practice, and scores below 80% of the total signified inadequate knowledge, a negative attitude, and inactive practice (16).

The electronic questionnaire method was implemented using the Questionnaire Star platform, generating a two-dimensional code for electronic questionnaire access. Participants logged in and completed the questionnaire by scanning the two-dimensional code provided through WeChat.

### Data analysis

Statistical analysis was executed using SPSS 26.0 (IBM Corp., Armonk, N.Y., USA). Continuous variables were depicted as mean ± standard deviation (SD), and compared by independent-samples *t-*tests or analysis of variance (ANOVA). Categorical variables were expressed as *n* (%). Pearson correlation analysis was conducted to examine the associations among knowledge, attitude, and practice scores. Multivariate and univariate logistic regression analyses were performed to explore the association between demographic characteristics using and KAP, with univariate variables with *P* < 0.05 were included in the multivariate regression. The 75% distribution of knowledge, attitude, and practice scores as cutoff for regression analyses (17). The structural equation modeling (SEM) was used to test the following hypothesis: (1) The knowledge had a direct impact on attitude; (2) The knowledge had a direct impact on practice; and (3) The attitude had a direct impact on practice. A two-sided *P* < 0.05 was considered as statistically significant.

### Ethical considerations

Ethical approval for the study was granted by the Medical Ethics Committee, and informed consent was duly obtained from all study participants.

### Validity and reliability

To ensure the quality and integrity of the questionnaire responses, a one-submission-per-IP address restriction was applied, and all questionnaire items were mandatory. Questionnaires with a short completion time and consistent response options were excluded during the statistical data cleaning process. Furthermore, the research team members meticulously reviewed all questionnaires to assess completeness, internal consistency, and overall validity.

## Results

### Characteristics of participants

A total of 482 parents were enrolled in this study. Demographic information from the respondents indicated that 379 (78.63%) were females, with an average age of 35.83 ± 6.40 years. Furthermore, 211 (43.78%) possessed junior college/undergraduate education or less, and 430 (89.21%) had children diagnosed with childhood acute lymphoblastic leukemia. The children had an average age of 6.72 ± 3.64 years, with an average disease duration of 16.10 ± 17.15 months, and 288 (59.75%) of the children commenced chemotherapy ≥6 months ago.

The mean knowledge, attitude, and practice scores were 7.28 ± 1.13 (possible range: 0–10), 37.82 ± 4.38 (possible range: 9–45), and 40.09 ± 4.17 (possible range: 9–45), respectively. The knowledge score displayed variation among parents of different genders (*P* < 0.001) and education (*P* = 0.004). Concerning the attitude score, differences were observed with regards to gender (*P* = 0.009), residence (*P* < 0.001), and monthly income (*P* < 0.001). Discrepancies in the practice score were identified among parents residing in different areas (*P* < 0.001), having varying education (*P* < 0.001), diverse monthly incomes (*P* < 0.001), and different types of children's diseases (*P* = 0.002). Furthermore, individuals with distinct occupations (*P* = 0.001, *P* = 0.003, *P* < 0.001, respectively), time since their child's treatment (*P* = 0.001, *P* = 0.021, *P* = 0.022, respectively), and diverse guardianships (*P* < 0.001, *P* = 0.028, *P* = 0.024, respectively) were likely to have varying knowledge, attitude, and practice scores (Table 1).

**Table 1:** The demographic characteristics and KAP scores.

**Characteristics**	**N (%)**	**Knowledge**		**Attitude**		**Practice**	
		**Mean** ±**SD**	**P**	**Mean** ±**SD**	**P**	**Mean** ±**SD**	**P**
**Total**	482	7.28 ± 1.13		37.82 ± 4.38		40.09 ± 4.17	
**Gender**			< 0.001		0.009		0.163
Male	103 (21.37)	6.93 ± 1.37		38.82 ± 4.17		40.60 ± 4.43	
Female	379 (78.63)	7.38 ± 1.04		37.55 ± 4.40		39.96 ± 4.09	
**Residence**			0.260		< 0.001		< 0.001
Rural	199 (41.29)	7.19 ± 1.21		37.19 ± 4.44		39.05 ± 4.53	
Urban	227 (47.10)	7.37 ± 1.06		38.66 ± 4.37		41.18 ± 3.73	
Suburban	56 (11.62)	7.25 ± 1.13		36.66 ± 3.56		39.41 ± 3.48	
**Education**			0.004		0.238		< 0.001
Middle school and below	156 (32.37)	7.04 ± 1.32		37.38 ± 4.40		38.81 ± 4.85	
High school and technical secondary school	115 (23.86)	7.35 ± 0.95		37.80 ± 4.04		39.35 ± 3.86	
Junior college and above	211 (43.78)	7.43 ± 1.05		38.16 ± 4.53		41.45 ± 3.32	
**Occupation**			0.001		0.003		< 0.001
Employed	152 (31.54)	7.54 ± 1.02		38.68 ± 4.64		41.58 ± 3.32	
Unemployed	330 (68.46)	7.16 ± 1.16		37.42 ± 4.20		39.41 ± 4.35	
**Monthly income, Yuan**			0.134		< 0.001		< 0.001
< 2,000	114 (23.65)	7.08 ± 1.36		36.38 ± 4.06		39.31 ± 4.49	
2,000–5,000	167 (34.65)	7.36 ± 0.98		37.87 ± 4.52		39.77 ± 4.35	
5,000–10,000	121 (25.10)	7.39 ± 1.07		38.33 ± 4.20		40.51 ± 3.94	
>10,000	80 (16.60)	7.25 ± 1.15		39.00 ± 4.30		39.31 ± 4.49	
**Types of children's diseases**			0.210		0.083		0.022
Childhood acute lymphoblastic leukemia	430 (89.21)	7.30 ± 1.12		37.94 ± 4.43		40.24 ± 4.07	
Other leukemia	52 (10.79)	7.10 ± 1.22		36.83 ± 3.83		38.85 ± 4.81	
**Time since child's treatment**			0.001		0.021		0.022
≤ 3 months	121 (25.10)	6.97 ± 1.37		37.12 ± 4.12		39.31 ± 4.49	
3–6 months	73 (15.15)	7.26 ± 1.04		37.21 ± 3.83		39.77 ± 4.35	
≥6 months	288 (59.75)	7.42 ± 1.02		38.27 ± 4.57		40.51 ± 3.94	
**Guardianship**			< 0.001		0.028		0.024
Father	103 (21.37)	6.93 ± 1.37		38.82 ± 4.17		40.60 ± 4.43	
Mother	369 (76.56)	7.40 ± 1.04		37.57 ± 4.38		40.04 ± 4.01	
Grandparents	10 (2.07)	6.70 ± 1.06		36.70 ± 5.31		36.90 ± 6.10	

The distribution of knowledge dimensions revealed that the three knowledge items with the highest accuracy rates were as follows: “To prevent nosebleeds in children, besides maintaining indoor humidity, advise the child not to pick their nose to avoid damaging the nasal mucosa” (K3) at 98.34%, “If a child is undergoing chemotherapy, the home should be kept clean with good ventilation. Fresh flowers or potted plants should be temporarily avoided indoors. Garbage bins should have lids, and garbage should not be stored for more than 2 h” (K1) at 98.13%, and “Children undergoing asparaginase treatment should be cautious about the occurrence of severe pancreatitis and maintain a light diet, avoiding high-fat and oily foods” (K10) at 97.51%. The three items with the lowest accuracy rates were “For children in the maintenance phase and those who have completely stopped taking medication, they should engage in moderate to high-intensity exercise for over an hour each day, such as brisk walking, cycling, jogging, aerobic exercises, etc.” (K7) at 63.69%, “If a child has a fever, they can take acetaminophen, ibuprofen, aspirin, compound aminopyrine, indomethacin, etc.” (K6) at only 6.43%, and “Children with leukemia should not receive any vaccinations during chemotherapy” (K9) at only 2.07% (Supplementary Table 1).

Parents exhibit varying attitudes toward their children's treatment, with only 21.57% not experiencing feelings of confusion and helplessness concerning the intricacies of their children's treatment (A2). A substantial parents (83.61%) expressed the ability to appropriately cope with their child's frustration during the treatment process (A4), while 89.63% remained confident in their ability to overcome their child's illness (A6). Furthermore, 86.72% expressed confidence in maintaining a composed disposition and facing the situation positively (A7). Additionally, 72.61% were resolute in their belief that their child should receive treatment at a specialized children's hospital to ensure standardized care (A8) (Supplementary Table 2).

Parents demonstrated varying levels of engagement in supporting their children's treatment, with 78.63% and 84.23%, respectively, consistently attending their child's medical reviews (P1) and encouraging timely medication adherence (P2). A notable 68.67% were adept at promptly recognizing their child's discomfort and effectively communicating with the doctor (P3). Ensuring that the child maintained a nutritious and hygienic diet (P4) was consistently upheld by 63.28% of parents, while 62.03% consistently provided effective oral care for their child (P5). Furthermore, 59.54% of parents consistently gathered and preserved detailed treatment-related information immediately for an extended duration (P6). Of concern is the fact that 48.55% of parents expressed concerns about having inadequate financial resources to support their child's treatment (P9) (Supplementary Table 3).

Correlation analysis revealed a strong positive association between knowledge and attitude (r = 0.17, *P* < 0.001) and practice (r = 0.25, *P* < 0.001). Additionally, a noteworthy positive correlation was observed between attitude and practice (r = 0.54, *P* < 0.001) (Table 2). Multivariate logistic regression analysis revealed that unemployment (OR = 0.59, 95% CI: [0.37–0.96], *P* = 0.033) and the duration of the child's illness (OR = 1.02, 95% CI: [1.00–1.03], *P* < 0.001) were independently associated with knowledge (Table 3). Meanwhile, the knowledge score (OR = 1.23, 95% CI: [1.00–1.50], *P* = 0.044) and residing in a rural area (OR = 0.50, 95% CI: [0.28–0.87], *P* = 0.014) were independently associated with attitude (Table 4). Additionally, the knowledge score (OR = 1.48, 95% CI: [1.08–2.05], *P* = 0.016), attitude score (OR = 1.31, 95% CI: [1.18–1.46], *P* < 0.001), education of junior college and above (OR = 11.28, 95% CI: [1.94–65.65], *P* = 0.007), and a monthly income of 5,000–10,000 Yuan (OR = 10.88, 95% CI: [1.15–102.98], *P* = 0.037) were independently associated with practice (Table 5).

**Table 2:** Correlation analysis.

	**Knowledge**	**Attitude**	**Practice**
Knowledge	1		
Attitude	0.17 (*P < * 0.001)	1	
Practice	0.25 (*P < * 0.001)	0.54 (*P < * 0.001)	1

**Table 3:** Univariate and multivariate analysis for knowledge.

**Characteristics**	**Univariate analysis**		**Multivariate analysis**	
	**OR (95%CI)**	**P**	**OR (95%CI)**	***P***
**Gender**				
Male	0.53 (0.34–0.82)	0.004		
Female	ref			
**Age, years**	0.98 (0.95–1.00)	0.096		
**Residence**				
Rural	0.75 (0.50–1.09)	0.132		
Urban	ref			
Suburban	0.75 (0.41–1.34)	0.327		
**Education**				
Middle school and below	ref		ref	
High school and technical secondary school	1.28 (0.79–2.08)	0.310	1.17 (0.71–1.94)	0.534
Junior college and above	1.90 (1.25–2.89)	0.003	1.58 (0.96–2.59)	0.071
**Occupation**				
Employed	ref		ref	
Unemployed	0.53 (0.35–0.78)	0.002	0.59 (0.37–0.96)	0.033
**Monthly income, Yuan**				
< 2,000	ref			
2,000–5,000	1.26 (0.78–2.03)	0.348		
5,000–10,000	1.42 (0.85–2.38)	0.182		
>10,000	1.29 (0.72–2.28)	0.391		
**Duration of the child's diseases (months)**	1.02 (1.00–1.03)	0.004	1.02 (1.00–1.03)	0.013
**Types of children's diseases**				
Childhood acute lymphoblastic leukemia	1.26 (0.71–2.25)	0.427		
Other leukemia	ref			
**Time since child's treatment**				
≤ 3 months	ref		ref	
3–6 months	1.33 (0.74–2.38)	0.335	1.21 (0.66–2.22)	0.543
≥ 6 months	1.70 (1.11–2.60)	0.015	1.18 (0.71–1.96)	0.525
**Child's age, years**	1.01 (0.96–1.06)	0.726		
**Guardianship**				
Father	0.51 (0.33–0.80)	0.003	0.41 (0.26–0.66)	< 0.001
Mother	ref		ref	
Grandparents	0.29 (0.08–1.15)	0.079	0.39 (0.10–1.60)	0.191

**Table 4:** Univariate and multivariate analysis for attitude.

**Characteristics**	**Univariate analysis**		**Multivariate analysis**	
	**OR (95%CI)**	**P**	**OR (95%CI)**	**P**
**Knowledge score**	1.25 (1.03–1.52)	0.027	1.23 (1.00–1.50)	0.044
**Gender**				
Male	1.54 (0.78–3.06)	0.215		
Female	ref			
**Age, years**	0.99 (0.95–1.03)	0.549		
**Residence**				
Rural	0.48 (0.27–0.83)	0.009	0.50 (0.28–0.87)	0.014
Urban	ref		ref	
Suburban	0.59 (0.26–1.36)	0.213	0.60 (0.26–1.39)	0.235
**Education**				
Middle school and below	ref			
High school and technical secondary school	1.72 (0.87–3.41)	0.122		
Junior college and above	1.69 (0.96–3.00)	0.071		
**Occupation**				
Employed	ref			
Unemployed	0.60 (0.33–1.09)	0.094		
**Monthly income, Yuan**				
< 2,000	ref			
2,000–5,000	1.67 (0.88–3.16)	0.118		
5,000–10,000	1.79 (0.88–3.63)	0.108		
>10,000	1.77 (0.79–3.96)	0.165		
**Duration of the child's diseases (months)**	1.02 (1.00–1.03)	0.109		
**Types of children's diseases**				
Childhood acute lymphoblastic leukemia	1.47 (0.70–3.08)	0.310		
Other leukemia	ref			
**Time since child's treatment**				
≤ 3 months	ref			
3–6 months	1.32 (0.60–2.90)	0.485		
≥6 months	1.64 (0.93–2.91)	0.089		
**Guardianship**				
Father	1.50 (0.75–2.97)	0.250		
Mother	ref			
Grandparents	0.42 (0.11–1.66)	0.215		

**Table 5:** Univariate and multivariate analysis for practice.

**Characteristics**	**Univariate analysis**		**Multivariate analysis**	
	**OR (95%CI)**	***P***	**OR (95%CI)**	***P***
**Knowledge score**	1.80 (1.41–2.29)	< 0.001	1.48 (1.08–2.05)	0.016
**Attitude score**	1.30 (1.18–1.43)	< 0.001	1.31 (1.18–1.46)	< 0.001
**Gender**				
Male	0.80 (0.36–1.76)	0.581		
Female	ref			
**Age, years**	0.95 (0.91–1.00)	0.030	1.01 (0.93 1.08)	0.906
**Residence**				
Rural	0.30 (0.14–0.66)	0.003	2.07 (0.68–6.25)	0.199
Urban	ref		ref	
Suburban	0.73 (0.19–2.79)	0.645	1.34 (0.28–6.49)	0.719
**Education**				
Middle school and below	ref		ref	
High school and technical secondary school	3.09 (1.29 7.39)	0.011	2.22 (0.71–6.91)	0.170
Junior college and above	13.87 (4.11–46.74)	< 0.001	11.28 (1.94–65.65)	0.007
**Occupation**				
Employed	ref		ref	
Unemployed	0.18 (0.06–0.60)	0.005	1.00 (0.20–4.86)	0.996
**Monthly income, Yuan**				
< 2,000	ref		ref	
2,000–5,000	2.03 (0.98–4.18)	0.056	0.85 (0.34–2.12)	0.715
5,000–10,000	24.00 (3.16–182.52)	0.002	10.88 (1.15–102.98)	0.037
>10,000	15.80 (2.07–120.65)	0.008	8.80 (0.76–102.18)	0.082
**Duration of the child's diseases (months)**	1.02 (0.99–1.05)	0.134		
**Types of children's diseases**				
Childhood acute lymphoblastic leukemia	2.61 (1.12–6.08)	0.026	2.07 (0.63–6.76)	0.231
Other leukemia	ref			
**Time since child's treatment**				
≤ 3 months	ref		ref	
3–6 months	1.46 (0.54–3.99)	0.459	0.95 (0.27–3.36)	0.940
≥ 6 months	2.22 (1.05–4.72)	0.037	1.69 (0.66–4.30)	0.273
**Child's age, years**	0.91 (0.84–0.99)	0.024	0.98 (0.86–1.12)	0.763
**Guardianship**				
Father	0.73 (0.33–1.62)	0.433	0.70 (0.23–2.09)	0.519
Mother	ref		ref	
Grandparents	0.16 (0.04–0.67)	0.012	0.35 (0.03–4.21)	0.411

The results of the SEM highlighted a significant direct impact of knowledge on attitude (β = 0.72, *P* = 0.002), attitude on practice (β = 0.57, *P* < 0.001), and knowledge on practice (β = 0.81, *P* < 0.001) (Table 6).

**Table 6:** SEM results.

			**Estimate**	***P***
Attitude	< —	Knowledge	0.72	0.002
Practice	< —	Attitude	0.57	< 0.001
Practice	< —	Knowledge	0.81	< 0.001
K1	< —	Knowledge	0.49	< 0.001
K2	< —	Knowledge	0.57	< 0.001
K3	< —	Knowledge	0.47	< 0.001
K4	< —	Knowledge	1.00	
K5	< —	Knowledge	0.41	< 0.001
K6	< —	Knowledge	0.02	0.774
K7	< —	Knowledge	0.89	< 0.001
K8	< —	Knowledge	0.37	< 0.001
K9	< —	Knowledge	0.04	0.447
K10	< —	Knowledge	0.45	< 0.001
A9	< —	Attitude	0.51	< 0.001
A8	< —	Attitude	0.44	< 0.001
A7	< —	Attitude	0.97	< 0.001
A6	< —	Attitude	0.89	< 0.001
A5	< —	Attitude	0.87	< 0.001
A4	< —	Attitude	1.00	
A3	< —	Attitude	0.82	< 0.001
A2	< —	Attitude	0.22	0.008
A1	< —	Attitude	0.68	< 0.001
P1	< —	Practice	0.42	< 0.001
P2	< —	Practice	0.31	< 0.001
P3	< —	Practice	0.61	< 0.001
P4	< —	Practice	0.59	< 0.001
P5	< —	Practice	0.60	< 0.001
P6	< —	Practice	0.81	< 0.001
P7	< —	Practice	0.90	< 0.001
P8	< —	Practice	1.00	
P9	< —	Practice	0.71	< 0.001

The result also indicated a mediating effect of attitude in the association between knowledge and practice. The total effect of knowledge on practice was significant (β = 1.22, 95% CI: 0.69–2.76, *P* = 0.004), with a significant direct effect (β = 0.81, 95% CI: 0.28–1.80, *P* = 0.022) and a significant indirect effect (β = 0.41, 95% CI: 0.20–0.79, *P* = 0.005). The indirect effect accounted for 33.80% of the total effect (Supplementary Table 4).

## Discussion

Parents of children with leukemia demonstrated inadequate knowledge, but positive attitude, and proactive practices toward family-based treatment for leukemia. While the study emphasized the constructive attitudes and active engagement of parents, it underscored the need for enhanced knowledge through educational initiatives and increased support, with a focus on developing both knowledge and practical parenting practices, aiming to optimize family-based treatments for leukemia.

The study uncovered that parent of children with leukemia exhibited limited knowledge while predominantly maintaining a positive attitude and demonstrating proactive practices in family-based treatment. These findings suggest the potential for enhancing parental comprehension of the disease and its management, particularly in a clinical context. To address this, targeted educational interventions can be devised to augment parents' knowledge while sustaining their positive attitudes and proactive practices, thereby contributing to improved clinical outcomes for children with leukemia (18, 19).

The study unveils significant variability in parental KAP related to family treatment for childhood leukemia. While the findings align with existing literature in part, particularly regarding disparities in knowledge, the notably positive attitudes and proactive practices observed here differ from some prior studies. These differences could potentially be attributed to the unique cultural and social context of the studied population (15). The data underscore the importance of customizing interventions to specific aspects of parental KAP (20–22). The influence of gender and education on knowledge scores underscores the necessity for educational interventions, especially for subgroups with lower educational attainment (23). Meanwhile, the impact of residence and income on attitudes suggests the requirement for location-specific and financial support strategies (24–26). The multifaceted factors influencing practice emphasize the need for a comprehensive approach to enhancing clinical practice, encompassing educational, financial, and psychosocial support components (27, 28).

The study's findings shed light on the intricate landscape of parental knowledge concerning the management of childhood leukemia. It reveals a commendable understanding in certain areas coexisting with significant knowledge gaps in others. Notably, the observed deficiencies in knowledge, particularly in aspects such as fever management, dietary precautions, and exercise misconceptions, underscore the pressing need to equip parents with accurate information. These efforts are essential for enhancing their role in clinical practice (29, 30). Such interventions play a pivotal role in optimizing the care provided to children with leukemia, fostering informed decision-making, and ultimately contributing to improved clinical outcomes. Furthermore, the unexplained variability in knowledge gaps necessitates in-depth investigation to unveil the underlying factors, thereby paving the way for more effective and culturally sensitive educational strategies.

The study's results indicate that parents of children with leukemia generally possess a positive attitude toward family-based treatment for leukemia. The majority of parents expressed agreement or strong agreement with statements that reflect their understanding of the treatment process, their confidence in post-discharge care, their resilience in facing setbacks, and their commitment to maintaining physical health. These positive attitudes are encouraging and align with the objective of cultivating a supportive and empowered caregiver role for parents within the context of childhood leukemia treatment (31, 32). Nevertheless, it is crucial to acknowledge that while the majority holds a positive attitude, there may still be a subset of parents facing challenges in this regard. Therefore, the findings underscore the importance of providing comprehensive psychosocial support and resources to ensure that all parents are well-prepared and confident in managing their child's leukemia treatment. This, in turn, contributes to improved clinical practice and the overall wellbeing of families in this situation (33).

The study's findings demonstrate that parents of children with leukemia generally engage in proactive practices within the domain of family treatment, aligning with the objective of empowering them to become active partners in care. A substantial majority of parents exhibit strong capabilities in various caregiving aspects, including managing appointments, overseeing medication regimens, and promptly addressing their child's discomfort, thus fostering a constructive synergy between parents and clinical care. However, the findings underscore a crucial aspect: the financial challenges faced by a subset of parents in meeting the expenses of their child's treatment. These challenges can introduce disparities in caregiving capacities, potentially limiting the active involvement of some parents. Addressing this financial burden is imperative for comprehensive clinical practice, and it necessitates proactive measures, such as providing information on available financial aid resources, healthcare subsidies, and insurance coverage. Ensuring equitable access to these provisions is essential in promoting not only the active role of all caregivers but also the comprehensive wellbeing of families dealing with childhood leukemia (34). Future interventions could involve structured coaching programs and educational groups that provide parents with practical caregiving techniques, such as managing daily routines, supporting their child's emotional needs, and recognizing early symptoms requiring medical attention.

The study's findings elucidate the intricate relationships among knowledge, attitude, and practice in the context of childhood leukemia care, underscoring the pivotal role of knowledge in shaping attitudes and influencing proactive practices. These findings align with other previous KAP research and underscore the need for tailored educational interventions to equip parents with the necessary information while fostering positive attitudes and encouraging proactive caregiving practices (14, 35). A noteworthy aspect is the robust connection between attitude and practice, suggesting that a positive attitude can serve as a potent catalyst for active engagement in caregiving (36). This finding highlights the emotional and psychological dimensions of parental caregiving in the context of childhood leukemia.

### Strengths and limitations of the work

This study presents certain limitations that warrant consideration. It employs a cross-sectional design, which restricts the establishment of causality and the identification of long-term trends. The reliance on self-administered questionnaires introduces potential response bias, notably the risk of social desirability bias, which may lead to an overestimation of positive attitudes and proactive practices. Lastly, the study's exclusive focus on knowledge, attitude, and practice omits the examination of potential psychosocial and healthcare system-related factors that might influence parental KAP. Additionally, the sample is not homogeneous, with more mothers participating than fathers, highlighting a gender imbalance. Besides, we used inter-rater reliability to ensure consistency during questionnaire development, but we did not conduct test-retest reliability, which could have helped check the stability of responses over time. Future studies should aim to involve more fathers and consider the couple's combined knowledge, rather than focusing on only one caregiver. The study also did not evaluate the type and frequency of communication between healthcare professionals and parents, nor whether healthcare teams were supported by psychologists. Furthermore, gathering information on support from other specialized or non-specialized individuals who may assist in caregiving would be beneficial. It is also possible that positive attitudes and practices may be underestimated due to comorbid psychological symptoms, such as depression, anxiety, or PTSD, which can influence caregiving behaviors. These factors could serve as moderating variables affecting attitudes and practices and warrant consideration in future studies to enhance understanding of the determinants influencing parental KAP.

### Recommendations for further research

Future research should consider longitudinal studies to track changes in knowledge, attitudes, and practices over time, offering insights into how these factors evolve throughout the different stages of leukemia treatment. Intervention studies are also recommended to assess the effectiveness of targeted educational programs designed to fill knowledge gaps identified in this study, such as management of fever, dietary precautions, and exercise misconceptions. Expanding research to include more diverse populations—encompassing various socioeconomic statuses and geographic locations—could help understand cultural and social influences on parental KAP. Additionally, exploring the psychosocial factors that influence parental attitudes and practices would be beneficial for designing supportive interventions. Lastly, it is crucial to investigate healthcare system influences, such as provider communication styles and resource availability, to understand how systemic changes might improve engagement and outcomes.

### Implications for policy and practice

Policymakers and healthcare institutions should develop and support educational programs that enhance parental knowledge about childhood leukemia. These programs need to be accessible and tailored to suit parents with various education levels and literacy. Creating structured support networks that offer continuous guidance and counseling can help sustain positive attitudes and proactive practices among parents. Such networks could include peer support groups, workshops, and online resources. Furthermore, there is an urgent need for financial assistance policies to help families manage the economic demands of treatment. Implementing subsidies, grants, or insurance modifications could alleviate financial burdens, allowing families to focus on caregiving. Healthcare practices should also integrate family-based care models into standard care protocols, recognizing the critical role families play in the treatment process and ensuring their needs are systematically addressed. Additionally, advocacy efforts should promote inclusive care policies that ensure all families, regardless of socio-economic status or location, receive the necessary support and resources for active engagement in the treatment process.

## Conclusion

In conclusion, parents of children with leukemia demonstrated inadequate knowledge, a predominantly positive attitude, and proactive practices toward family-based treatment for leukemia. While the study emphasized the constructive attitudes and active engagement of parents, it underscored the need for enhanced knowledge through educational initiatives and increased support, particularly by focusing on improving knowledge and parenting practices to optimize family-based treatments for leukemia.

## Data Availability

The original contributions presented in the study are included in the article/Supplementary material, further inquiries can be directed to the corresponding author.
