# Supplementary material for: Knowledge, attitude, and practice toward family-based treatment among parents of children with leukemia
Source: Front Public Health. 2024 Nov 25;12:1481122. doi: 10.3389/fpubh.2024.1481122 (PMC11625665; doi:10.3389/fpubh.2024.1481122)
Supplement: Supplementary file 1 [file Table_1.pdf]

Supplementary Table 1. Distribution of knowledge

	Correctness N(%)
**1. If a child is undergoing chemotherapy, the home should be kept clean with good ventilation. Fresh flowers or potted plants should be temporarily avoided indoors. Garbage bins should have lids, and garbage should not be stored for more than 2 hours.**	473(98.13)
**2. Oral ulcers are a common side effect of chemotherapy and can affect a child's eating. You can prepare a saline solution by youself (baking soda + salt + water), or use mouthwash provided by the hospital, or mouthwash non-containing alcohol.**	438(90.87)
**3. To prevent nosebleeds in children, besides maintaining indoor humidity, advise the child not to pick their nose to avoid damaging the nasal mucosa.**	474(98.34)
**4. If a child has a nosebleed, make them sit upright and use their fingers to press on the ala nasi for 10 minutes.**	398(82.57)
**5. If a child's platelet count is low (generally less than 20×109/L), they are at risk of spontaneous bleeding. Therefore, it is necessary to avoid all sports with a strong impact (such as jumping, soccer, basketball, etc.).**	467(96.89)
**6. If a child has a fever, they can take acetaminophen, ibuprofen, aspirin, compound aminopyrine, indomethacin, etc.**	31(6.43)
**7. For children in the maintenance phase and those who have completely stopped taking medication, they should engage in moderate to high intensity exercise for over an hour each day, such as brisk walking, cycling, jogging, aerobic exercises, etc.**	307(63.69)
**8. When children receiving chemotherapy go outside, they can go to open spaces with good air circulation and lower population density.**	442(91.70)
**9. Children with leukemia should not receive any vaccinations during chemotherapy.**	10(2.07)
**10. Children undergoing asparaginase treatment should be cautious about the occurrence of severe pancreatitis and maintain a light diet, avoiding high-fat and oily foods.**	470(97.51)

Supplementary Table 2. Distribution of attitude

	Strongly agree	Agree	Neutral	Disagree	Strongly disagree
**1. The explanations from the medical staff are clear enough, and I fully understand the purpose of each treatment my child receives. (P)**	254(52.70)	189(39.21)	34(7.05)	3(0.62)	2(0.41)
**2. The treatment my child is receiving is too complicated, and I often feel lost and helpless. (N)**	74(15.35)	153(31.74)	151(31.33)	82(17.01)	22(4.56)
**3. After my child is discharged from the hospital, I have the confidence to take care of them. (P)**	225(46.68)	197(40.87)	56(11.62)	2(0.41)	2(0.41)
**4. I can correctly face the setbacks my child experiences during the treatment process. (P)**	193(40.04)	210(43.57)	70(14.52)	8(1.66)	1(0.21)
**5. When it comes to taking care of my child, I feel that my physical health is not a problem. (P)**	195(40.46)	201(41.70)	78(16.18)	7(1.45)	1(0.21)
**6. My child's illness is just a challenge on their life journey and can be overcome. (P)**	239(49.59)	93(40.04)	41(8.51)	7(1.45)	2(0.41)
**7. I can keep a calm mind and face the current situation positively. (P)**	196(40.66)	222(46.06)	60(12.45)	3(0.62)	1(0.21)
**8. It is essential to seek treatment at specialized children's hospitals for standardized care. (P)**	350(72.61)	126(26.14)	5(1.04)	0	1(0.21)

**9. For early treatment, the most important thing is to observe the child's response to chemotherapy, making timely adjustments to the treatment regimen, and adhere to the treatment. (P)**	327(67.84)	148(30.71)	5(1.04)	1(0.21)	1(0.21)

Supplementary Table 3. Distribution of practice

	Always	Often	Sometimes	Rarely	Never
**1. I can ensure that my child goes for follow-up appointments on time. (P)**	379(78.63)	97(20.12)	6(1.24)	0	0
**2. I can supervise my child to take their medications on schedule. (P)**	406(84.23)	74(15.35)	2(0.41)	0	0
**3. I can promptly notice any discomfort my child experiences and communicate with the doctor. (P)**	331(68.67)	131(27.18)	20(4.15)	0	0
**4. I can provide my child with a nutritionally rich diet and ensure hygiene. (P)**	305(63.28)	160(33.20)	17(3.53)	0	0
**5. I can assist my child with oral care. (P)**	299(62.03)	167(34.65)	14(2.90)	2(0.41)	0
**6. I will collect and organize detailed information immediately during or after the treatment and keep it for the long term. (P)**	287(59.54)	155(32.16)	35(7.26)	3(0.62)	2(0.41)
**7. I will obtain disease-related information and data from legitimate sources (e.g., through extensive communication with doctors). (P)**	278(57.68)	143(29.67)	49(10.17)	9(1.87)	3(0.62)
**8. I have convenient means to address difficulties and setbacks my child may face during treatment (e.g., outpatient services, online consultations, in-patient consultations). (P)**	224(46.47)	170(35.27)	58(12.03)	24(4.98)	6(1.24)
**9. I have sufficient financial support to meet the expenses of my child's treatment. (P)**	124(25.73)	124(25.73)	130(26.97)	92(19.09)	12(2.49)

Supplementary Table 4. The direct and indirect effects

Model paths			Total effects		Direct Effect		Indirect effect	
			β (95% CI)	P	β (95% CI)	P	β (95% CI)	P
A	<---	K	0.72 (0.36, 1.55)	0.004	0.72 (0.36, 1.55)	0.004	/	/
P	<---	K	1.22 (0.69, 2.76)	0.004	0.81 (0.28, 1.80)	0.022	0.41 (0.20, 0.79)	0.005
P	<---	A	0.57 (0.46, 0.71)	0.006	0.57 (0.46, 0.71)	0.006	/	/
